# Seroprevalence and risk factors *of Toxoplasma gondii* among pregnant women in Adwa district, northern Ethiopia

**DOI:** 10.1186/s12879-019-3936-0

**Published:** 2019-04-16

**Authors:** Mebrahtu Teweldemedhin, Amaha Gebremichael, Gebretsadkan Geberkirstos, Haftom Hadush, Tuam Gebrewahid, Solomon Weldegebreal Asgedom, Berihu Gidey, Negasi Asres, Hailay Gebreyesus

**Affiliations:** 1 Department of Medical Laboratory Sciences, College of Health Sciences, Aksum University, P.O.BOX: 298, Aksum, Ethiopia; 2 School of Pharmacy, College of Health Sciences, Mekelle University, Mekelle, Tigray Ethiopia; 3 Department of Public Health, College of Health Sciences, Aksum University, Aksum, Ethiopia

**Keywords:** Pregnant women, Risk factors, Seroprevalence, *Toxoplasma gondii*

## Abstract

**Background:**

*Toxoplasma gondii* infection is a great health concern to pregnant women and the developing fetus. The aim of this study was to determine the seroprevalence of *T. gondii* and its associated factors in Adwa district.

**Methods:**

A facility based cross-sectional study was conducted from January to June 2018 in Adwa district. Structured, a pre-tested questionnaire was used to collect the demographic and risk factor related data. Serum sample, collected from each of the study subjects was tested for IgG and IgM anti *T.godii* specific antibodies using Enzyme-Linked Immunosorbent Assay. A bivariable and multivariable logistic regression model was applied to show association between the dependent and independent variables considering *P* < 0.05 and the 95% confidence interval.

**Result:**

Out of the 360, 128 (35.6%) pregnant women were found to be positive for antibodies specific to *T. gondii*. Furthermore, 117 (32.5%) women were positive only for IgG, and 11 (3.1%) were positive both for IgM and IgG antibodies. Age, educational level, habit of hand washing after contact with garden soil or domestic animals, presence of domestic cat, history of contact with domestic dog and consumption of raw vegetables were significantly associated with *T. gondii*.

**Conclusion:**

The seroprevalence of *T. gondii* among pregnant women in the study area is low compared to the other regions of Ethiopia, and within the range of the seroprevalences in the central and East Africa region. However, efforts should be done to create awareness on the potential risk factors of the parasite in the community.

## Background

Toxoplasmosis is among the major global zoonotic diseases caused by *Toxoplasma gondii* (*T. gondii)* that infect almost all warm-blooded animals including humans [1]. Around 30% of the human population worldwide is chronically infected with *T. gondii* [2, 3]. The seroprevalence of toxoplasmosis ranged from 10 to 50% in developed countries and over 80% in developing tropical countries [4]. Globally, there are 190,100 new cases of congenital toxoplasmosis; 26, 500 (2/1000 live births) being in the Africa region [5]. Studies conducted in some parts of Ethiopia had also indicated that there was a high seroprevalence of *T. gondii* (74.73%) infection with higher odds of infection in pregnant women [6].

*T. gondii* infection causes severe and life threatening consequences in human beings and particularly it is a great health concern to pregnant women and the developing fetus or newborns [7]. In Ethiopia, about 82% women of childbearing age haveimmunity to *T. gondii* [8]; the rest may be at risk of developing an acute infection if exposed to the parasite. Maternal infection of *T. gondii* is associated with psychiatric disturbances and poor obstetric outcomes including abortion, still birth and psychomotor retardation. In women, infection was associated with anxiety, depression, schizophrenia spectrum disorders, and suicide attempts [9]. The risk of vertical transmission is lower during the first trimester (15–20%) than the third trimester of maternal infection (60–90%). However, severe fetal disease is associated with the first trimester of maternal infection [10, 11]; an infected fetus may has severe complications such as hearing problems, mental retardation, seizure, Chorioretinitis and hydrocephaly [12–14].

Changing environmental conditions, whether caused by rapid urbanization, global warming or economic globalization, have already influenced the emergence, transmission and distribution of toxoplasmosis [15]. The ecology of *T. gondii*, with its complex life-cycle, is susceptible to environmental changes mainly affecting the survival time and infectivity of oocysts, and the behavior and population density of hosts [16]. Giving antibiotic treatment is not the most effective way to prevent congenital toxoplasmosis and associated complications. Primary prevention is best [17]. Besides, clinical diagnosis of toxoplasmosis is difficult, considering that 90 to 95% of the infected pregnant women are asymptomatically infected [18] increasing the risk of congenital infection. Therefore, serological screening of *T. gondii* infection is essential to prevent the risk of congenital toxoplasmosis. Upon early screening, the use of drugs such as spiramycine was found to prevent congenital infection by more than 60% [2].

Ethiopia is one of the countries with low resources including scarcity of safe drinking water supply particularly in the rural communities; with different domestic animals including cats, dogs, goats, sheep and camel and climatic conditions favoring the survival of the parasite. Considering the above hosts and other predisposing factors for *T. gondii*, research is needed particularly among pregnant women to depict the prevalence of the parasite. Women engage in different home-based activities such as food preparation and caring their children; they are frequently exposed to domestic animals and garden soil. Hence, screening of pregnant women for *T. gondii* infection during their Maternal and Child Health (MCH) service is not only of benefit of the women but also benefit the next generation. However, in most parts of Ethiopia, including the study area, there is no continuous screening program for *T. gondii* because of facility limitations and cases are mostly diagnosed by exclusion. Therefore, the aim of this study was to determine the seroprevalence of *T. gondii* and its associated factors in Adwa district.

## Methods

### Study area, period and design

A facility based cross-sectional study was conducted from January to June 2018 in Adwa district, Central zone of Tigray, Northern Ethiopia, located at a distance of 1006 km from Addis Ababa (the capital city of Ethiopia). It is found at an elevation of 1907 m with a latitude and longitude of 14°10′N 38°54′E. The district has one general hospital and 3 health centers with a total catchment population of 1,670,001. Adwa is basically an urban dwelling but the hospital and health centers serve for women from urban dwelling as well as from nearby rural dwellings.

### Populations

All pregnant women in Adwa district were the source population; pregnant women who visited public hospitals and health centers in Adwa district comprised the study population.

### Sample size determination

The sample size was calculated based on the single proportion formula because it yielded a maximum sample size.

The sample size was calculated considering, he estimated seroprevalence (p) of *T. gondii* among pregnant women; 68.4% from the study conducted in Debre Tabor, Ethiopia [19]; considering the 95% confidence level, α is 0.05 (the level of significance) and the value of Z at α/ 2 is 1.96; d is the margin of error (5%). Therefore, based on these assumptions *n* = 332; with10% contingency, the final sample size was 365.

### Sampling technique

Out of the three zones in the catchment of Aksum University, Central zone was selected by lottery method; one district (Adwa) was selected again by lottery method. The total sample size was then proportionally allocated to health facilities in Adwa district (Adwa hospital, Adwa health center, Berhe Gebremedhn health center and Atsedemariam health center) based on their previous flow of pregnant women. Finally, pregnant women who were attending each health institution during the study period were randomly included in the study as per the allocated quota. Pregnant women in the age range of 15–49 years were included whereas pregnant women who were unable to communicate for medical reason were excluded from the study.

### Study variables

Independent variables: Age, occupation, residence, educational level, trimester of pregnancy, history of abortion, history of blood transfusion, history of needle stick injury, presence of domestic cat, consumption of raw meat, consumption of raw milk, consumption of raw vegetables, hand washing after contact with garden soil or domestic animals, source of drinking water, history of contact with sheep/goat, history of contact with camel and history of contact with dog. Dependent variable: Seroprevalence of *T. gondii.*

### Data collection procedures

#### Socio-demographic and related data

Structured, pretested questionnaires, translated in to Tigrigna (local language), were used to collect the demographic and risk factor related data. Recruited health professionals filled the questionnaire by direct interview of the study participants. Patients’ medical records were also used to confirm the clinical factors such as history of blood transfusion.

#### Specimen collection and processing

After completion of the questionnaire, trained laboratory technologists collected adequate specimen (paired 5-10 ml of venous blood) following aseptic techniques. The whole blood was centrifuged at 3000 rpm for 5 min to get serum sample; tested for IgG and IgM anti*T.godii* specific antibodies using Enzyme Linked Immunosorbent Assay (ELISA) as per the standard operating procedures. The serums samples were tested using Toxo IgG assay (sensitivity = 99.7%, specificity = 99.6%) and Toxo IgM assay (sensitivity = 99.95%) (ABBOT, ARCHITECT). Reactive serum samples for IgM and or IgG and with high IgG avidity were considered as past infections whereas reactive samples for IgM and IgG with low IgG avidity in the paired serum (second serum specimen taken after 3 weeks of the primary specimen) were considered as recent infections.

### Quality assurance

Strict measures were taken throughout the analytic process. Five percent of the Questionnaire was pre-tested among pregnant women attending at Suhul Hospital (Shire town), and Questionnaire was revised accordingly. Data collectors were trained for 2 days on how to conduct the interview and the sampling process. Completed questionnaires were reviewed immediately to ensure accuracy and legibility. Quality control samples were tested parallel with the research samples and standard operating procedures were followed during the laboratory investigation.

### Statistical analysis

Data were entered into Epiinfo version 3.5.3 and analysis was made using the statistical software SPSS version 21 for Windows. Logistic regression model was applied to show association between the dependent and independent variables. Firstly, bivariable logistic regression model was analyzed to see association between each of the independent and the outcome variable. The independent variables with a *P* value < 0.05 in the bivariable logistic regression were together entered to the multivariable logistic regression analysis to adjust potential confounders and analyzed using the enter method. Finally, statistically significant association has been declared considering a *P* value < 0.05 and the corresponding 95% confidence interval.

## Results

### Characteristics of the study subjects

This study included a total of 360 pregnant women with a response rate of 98.6%. Majority (65%) of them were urban dwellers and the mean age (± SD) of the study subjects were 26.9 (± 5.7) years (see Table 1).

**Table 1:** Socio-demographic characteristics, behavioral and clinical factors of the study subjects (*n* = 360)

Socio-demographic Variables	Categories	Frequency(n)	Percent (%)
Residence	Rural	126	35.0
	Urban	234	65.0
Age	15–24	117	32.5
	25–34	178	49.4
	>/=35	65	18.1
Occupational	Student	45	12.5
	House Wife	143	39.7
	Farmer	42	11.7
	Governmental employee	65	18.1
	Non-governmental employee	26	7.2
	Self employed	39	10.8
Educational status	Unable to read and write	47	13.1
	1–8	72	20.0
	9–12	135	37.5
	College and above	106	29.4
Trimester of pregnancy	First trimester	114	31.7
	Second trimester	130	36.
	Third trimester	116	32.1
Source of drinking water	Unprotected well	23	6.4
	protected well	78	21.7
	Tap water	259	71.9
Hand washing after contact with garden soil or domestic animals	Rarely	85	23.6
	Mostly	120	33.3
	Always	155	43.1
Domestic cat	No	186	13.1
	Yes	174	20.0
History contact with dog	No	272	75.6
	Yes	88	24.4
History of Contact with goat/sheep	No	285	79.2
	Yes	75	20.8
History of Contact with Camel	No	328	91.1
	Yes	32	8.9
Used to consume raw meat	No	255	70.8
	Yes	105	29.2
Used to consume raw milk	No	278	77.2
	Yes	82	22.8
Used to consume raw vegetables	No	128	35.6
	Yes	232	64.4
History of abortion	No	257	71.4
	Yes	103	28.6
History of needle stick injury	No	313	86.9
	Yes	47	13.1
History of blood transfusion	No	303	84.2
	Yes	57	15.8

### Seroprevalence of *T. gondii* and its associated factors

In this study, the serum sample was collected from a total of 360 pregnant women for the serological test, out of this, 128 (35.6%) were found to be positive for antibodies specific to *T. gondii*. Furthermore, 117 (32.5%) were found to be positive only for IgG; the rest 11 (3.1%) were positive both for IgM and IgG. Out of the 11 women with recent infection, 7 were in the first trimester, 3 were in the second trimester and only 1 mother was in the third trimester of pregnancy.

According to the bivariable logistic regression analysis, 12 variables including residence, age, occupation, educational level, trimester of pregnancy, source of drinking water, hand washing, presence of demotic cat, contact with dog, contact with domestic goat/sheep and consumption of raw vegetables were found to be significantly associated with seroprevalence of *T. gondii* (Table 2). However, after the Multivariate analysis, the statistically significant predictors were Age [AOR = 42.5, 95%CI:- 12.7–141.7), *P* < 0.01], educational level [AOR = 5.8, 95%CI:1.27–26.7, *P* = 0.02], habit of hand washing after contact with garden soil or domestic animals [AOR = 7.7, 95%CI:2.7–21.9), *P* < 0.01], presence of domestic cat [AOR = 5.2, 95%CI:2.36–11.6, *P* < 0.01], history of contact with domestic dog [AOR = 12.9, 95%CI:4.9–34.2, *P* < 0.01] and consumption of raw vegetables [AOR = 6.7, 95%CI:2.6–17.2, *P* < 0.01] (Table 3).

**Table 2:** Bivariate logistic regression analysis of factors associated with sero-prevalence of *T. gondii* among pregnant women (*n* = 360)

Variable	Sero-status	COR	95%CI	*P*-value
	Positive, n (%)			
Residence				
Rural	69 (54.8)	3.59	(2.270–5.679)	< 0.01
Urban	59 (25.2)	1		
Age				
15–24	13 (11.1)	1	1	
25–34	65 (36.5)	4.6	(2.8–8.8)	< 0.01
>/=35	50 (76.9)	26.6	(11.8–60.3)	< 0.01
Occupation				
Student	9 (20)	0.49	(0.2–1.2)	0.116
House wife	73 (51)	2.0	(1.1–3.7)	0.022
Farmer	11 (26.2)	0.7	(0.3–1.6)	0.4
Governmental employee	22 (33.8)	1	1	
Non-governmental employee	4 (15.4)	0.35	(0.1–1.16)	0.086
Self employed	9 (23.1)	0.58	(0.23–1.4)	0.24
Educational level				
Unable to read and write	21 (44.7)	2.25	(1.09–4.6)	0.03
1–8	39 (54.2)	3.3	(1.7–6.2)	< 0.01
9–12	40 (29.6)	1.17	(0.6–2.1)	0.5
College and above	28 (26.4)	1		
Trimester of pregnancy				
First trimester	41 (36.0)	0.88	(0.52–1..5)	0.65
Second trimester	42 (32.3)	0.75	(0.4–1.3)	0.28
Third trimester	45 (38.8)	1		
Source of drinking water				
Unprotected well	14 (60.9)	3.5	(1.5–8.5)	< 0.01
Protected well	35 (44.9)	1.8	(1.1–3.1)	0.02
Tap water	79 (30.5)	1		
Hand washing after contact with garden soil and/or domestic animals				
Rarely	48 (56.5)	2.8	(1.6–5.0)	< 0.01
Mostly	32 (26.7)	0.811	(0.5–1.4)	0.436
Always	48 (31)	1		
Presence of domestic cat				
No	42 (22.6)	1		
Yes	86 (49.4)	3.35	(2.13–5.3)	< 0.01
History contact with dog				
No	80 (29.4)	1		
Yes	48 (54.5)	2.8	(1.76–4.7)	< 0.01
History of contact with goat/sheep				
No	91 (31.9%)	1		
Yes	37 (49.3)	2.076	(1.24–3.5)	0.01
History of Contact with Camel				
No	113 (34.5)	1		
Yes	15 (46.9)	1.67	(0.8–3.5)	0.16
Used to consume raw meat				
No	86 (33.7)	1		
Yes	42 (40.0)	1.31	(0.8–2.09)	0.25
Used to consume raw vegetables				
No	26 (20.3)	1		
Yes	102 (44.0)	3.078	(1.8–5.08)	< 0.01
Used to consume raw milk				
No	98 (35.3)	1		
Yes	30 (36.6)	1.06	(0.6–1.7)	0.8
History of abortion				
No	90 (35.0)	1		
Yes	38 (36.9)	1.08	(0.6–1.7)	0.7
History of needlestick injury				
No	110 (35.1)	1		
Yes	18 (38.3)	1.14	(0.6–2.15)	0.67
History of blood transfusion				
No	111 (36.6)	1		
Yes	17 (29.8)	0.7	(0.4–1.35)	0.3

Note: *CI* confidence interval, *COR* Crude Odds Ratio; 1: referent

**Table 3:** Multivariate logistic regression analysis of factors associated with seroprevalence of *T. gondii* among pregnant women (*n* = 360)

Variable	Sero-status, n (%)	COR (95%CI), *P*-value	AOR (95%CI), *P*-value
Positive, n(%)			
Residence			
Rural	69 (54.8)	3.6 (2.3–5.7), < 0.01	1.6 (0.49–5.6),0.41
Urban	59 (25.2)	1	1
Age			
15–24	13 (11.1)	1	
25–34	65 (36.5)	4.6 (2.4–8.8), < 0.01	15.6 (5.9–40.9),< **0.01**
>/=35	50 (76.9)	26.6 (11.8–60.3),< 0.01	42.5 (12.7–141.7),< **0.01**
Occupation			
Student	9 (20)	0.49 (0.2–1.2), 0.1	0.4 (0.08–2.01),0.27
House Wife	73 (51)	2 (1.1–3.7), 0.02	0.62 (0.18–2.1),0.45
Farmer	11 (26.2)	0.7 (0.3–1.6)	0.64 (0.17–2.3), 0.51
Governmental employee	22 (33.8)	1	1
Non-governmental employee	4 (15.4)	0.35 (0.1–1.16), 0.08	0.21 (0.03–1.3),0.09
Self employed	9 (23.1)	0.58 (0.23–1.4), 0.24	0.5 (0.12–2.47),0.4
Educational level			
Unable to read and write	21 (44.7)	2.25 (1.09–4.6), 0.03	5.8 (1.27–26.7), **0.02**
1–8	39 (54.2)	3.3 (1.7–6.2),< 0.01	2.9 (0.7–11.7),0.13
9–12	40 (29.6)	1.17 (0.6–2.1), 0.5	3.2 (1.09–9.1),**0.03**
College and above	28 (26.4)	1	1
Source of drinking water			
Unprotected well	14 (60.9)	3.5 (1.5–8.5), < 0.01	1.3 (0.24–7.18),0.7
Protected well	35 (44.9)	1.8 (1.1–3.1), 0.02	0.6 (0.2–1.80),0.4
Tap water	79 (30.5)	1	1
Hand washing after contact with garden soil or domestic animals			
Rarely	48 (56.5)	2.8 (1.6–5.0)	7.7 (2.7–21.9),< **0.01**
Mostly	32 (26.7)	0.8 (0.5–1.4), 0.4	0.46 (02–1.06),0.07
Always	48 (31)	1	1
Presence of domestic cat			
No	42 (22.6)	1	1
Yes	86 (49.4)	3.35 (2.13–5.3), < 0.01	5.2 (2.36–11.6), < **0.01**
History contact with dog			
No	80 (29.4)	1	1
Yes	48 (54.5)	2.8 (1.7–4.7), < 0.01	12.9 (4.9–34.2), < **0.01**
History of contact with goat/sheep			
No	91 (31.9)	1	1
Yes	37 (49.3)	2.07 (1.2–3.5), 0.01	1.5 (0.5–4.7), 0.4
History of Contact with Camel			
No	113 (34.5)	1	1
Yes	15 (46.9)	1.67 (0.8–3.5), 0.16	0.5 (0.18–1.6), 0.29
Used to consume raw meat			
No	86 (33.7)	1	1
Yes	42 (40.0)	1.3 (0.8–2.09),0.25	1.7 (0.8–3.5), 0.17
Used to consume raw vegetables			
No	26 (20.3)	1	1
Yes	102 (44)	3.07 (1.8–5.08), < 0.01	6.7 (2.6–17.2), < **0.01**
Used to consume raw milk			
No	98 (35.3)	1	1
Yes	30 (36.6)	1.06 (0.6–1.7),0.8	2.05 (0.86–4.8), 0.1
History of abortion			
No	90 (35.0)	1	1
Yes	38 (36.9)	1.08 (0.6–1.7), 0.7	0.8 (0.36–1.7), 0.6
History of needlestick injury			
No	110 (35.1)	1	1
Yes	18 (38.3)	1.14 (0.6–2.15), 0.67	2.8 (0.9–8.9), 0.07
History of blood transfusion			
No	111 (36.6)	1	1
Yes	17 (29.8)	0.7 (0.4–1.35), 0.3	0.29 (0.07–1.07), 0.6

Note: *AOR* Adjusted Odds Ratio, *CI* confidence interval, *COR* Crude Odds Ratio; 1: referent; variable with a *p* value presented in boldface are statistically significant

## Discussion

Little was known about the seroprevalence of *T. gondii* in Tigray region and this is the first report of *T. gondii* infection in Adwa, Ethiopia. The seroprevalence of *T. gondii* among pregnant women in the study area was found to be 35.6%. This finding is lower than the findings from Arbaminch, Ethiopia 79.3% [20], Bench Maji, Ethiopia 85.3% [21], Brazil 68.37% [22], Ghana 51.2% [23] but higher than the findings in Tanzania 30.9% [24], Sri Lanka 30.27% [25], China 18.1% [26] and Mexico 11% [27]. The current finding is also lower than the seroprevalence of *T. gondii* in the lower altitude and higher humidly cities of Kenya, Kisumu (52%) and Mombasa (57%) [28]. Among the seropositive women, the majority of them were found to have a chronic or past infection. However, eleven women (11/128, 8.5%) or 3.1% of the 360 women was found to have a recent infection which is almost comparable to the studies conducted in other regions of Ethiopia [20, 21]. Considering the asymptomatic nature of the disease and the possibility of congenital spread, the serologic finding of this study should not be ignored. This is because the healthcare facilities in the study area lack specific tests for *T. gondii;* diagnosis is made by exclusion which may result in misdiagnosis or delayed diagnosis.

In this study, different risk factors have been assessed for the seroprevalence of *T. gondii*. Accordingly, the risk of contracting *T. gondii* had increased with age of the pregnant women; increased odds of having *T. gondii* infection was observed in the age group of greater than 24 years as compared to pregnant women in the age group of 15–24 years. This finding is consistent with the studies conducted in Tanzania [24] and Mali [29]. The seroprevalence of *T. gondii* associated with increasing maternal age might be attributed to the increased risk of contracting infection (7%) for 1 year increase of maternal age [7]. Pregnant women who were unable to read and write were 5.8 times more likely to be infected as compared to those who had at least college education; however, this might be due to lack of awareness of the potential risks, proximity to domestic animals, and gardening. The odds of having *T. gondii* were 7.7 times higher among pregnant women who rarely wash their hands after contact with garden soil and or domestic animals as compared to those women who always wash their hands. Those pregnant women with a history of contact with a domestic dog and those who used to consume raw vegetables had 12.9 and 6.7 times increased odds of *T. gondii* infection respectively as compared to their counterparts. In addition, pregnant women who own domestic cat had 5.2 times higher odds of being seropositive for *T. gondii*. Supporting these findings, contact with garden soil, owing to a domestic dog or cat, and consumption of raw vegetables was significantly associated with *T. gondii* infection in other studies conducted in Ethiopia [20, 21, 30], China [31], and Brazil [32].

Cats and dogs are thought to be the important amplifiers of infection of *T. gondii* [33, 34]. In the Ethiopian context, it is common to see domestic cats live and sleep together with human beings. Cats can directly contaminate humans, other animals, and their surrounding through their feces [7]. Likewise, after contact with cat’s feces, dogs can contribute to the transmission of the organism through mechanical contamination of garden soil, vegetables, and human beings. Hence, human beings can possibly acquire the infection or ingest the oocyst of the parasite through their contaminated hands after direct contact with cats or dogs, garden soil, and surfaces or consumption of contaminated vegetables. Oocysts of *T. gondii* are tough free-living stages of the parasite, and therefore are a major contributor to infections associated with the aforementioned risk factors because they [35]. On the other hand, dogs may acquire the infection from other warm-blooded animals through carnivore contamination, and in turn infect human beings [36].

## Conclusion

In conclusion, the seroprevalence of *T. gondii* among pregnant women in the study area is low compared to the other regions of Ethiopia, and within the range of the seroprevalences in the central and East Africa region. The main risk factors were increasing maternal age, educational level, lack of hand washing after contact with garden soil or domestic animals, the presence of a domestic cat, history of contact with domestic dog and consumption of raw vegetables. Efforts should be done to create awareness on the potential risk factors of the parasite in the community.

## Abbreviations

ELISA: Enzyme Linked Immunosorbent Assay
IgG: Immunoglobulin G
IgM: Immunoglobulin M
MCH: Maternal and Child Health
